# One’s trash is someone else’s treasure: sequence read archives from Lepidoptera genomes provide material for genome reconstruction of their endosymbionts

**DOI:** 10.1186/s12866-022-02602-1

**Published:** 2022-08-30

**Authors:** Victoria G. Twort, Daniel Blande, Anne Duplouy

**Affiliations:** 1 Finnish Natural History Museum, LUOMUS, The University of Helsinki, Helsinki, Finland; 2 Organismal and Evolutionary Biology, The University of Helsinki, Helsinki, Finland

**Keywords:** Lepidoptera, Symbionts, Trace archives, Metagenomes, *Wolbachia*, *Spiroplasma*

## Abstract

**Background:**

Maternally inherited bacterial symbionts are extremely widespread in insects. They owe their success to their ability to promote their own transmission through various manipulations of their hosts’ life-histories. Many symbionts however very often go undetected. Consequently, we have only a restricted idea of the true symbiont diversity in insects, which may hinder our understanding of even bigger questions in the field such as the evolution or establishment of symbiosis.

**Results:**

In this study, we screened publicly available Lepidoptera genomic material for two of the most common insect endosymbionts, namely *Wolbachia* and *Spiroplasma,* in 1904 entries, encompassing 106 distinct species. We compared the performance of two screening software, Kraken2 and MetaPhlAn2, to identify the bacterial infections and using a baiting approach we reconstruct endosymbiont genome assemblies. Of the 106 species screened, 20 (19%) and nine (8.5%) were found to be infected with either *Wolbachia* or *Spiroplasma*, respectively. Construction of partial symbiotic genomes and phylogenetic analyses suggested the *Wolbachia* strains from the supergroup B were the most prevalent type of symbionts, while *Spiroplasma* infections were scarce in the Lepidoptera species screened here.

**Conclusions:**

Our results indicate that many of the host-symbiont associations remain largely unexplored, with the majority of associations we identify never being recorded before. This highlights the usefulness of public databases to explore the hidden diversity of symbiotic entities, allowing the development of hypotheses regarding host-symbiont associations. The ever-expanding genomic databases provide a diverse databank from which one can characterize and explore the true diversity of symbiotic entities.

**Supplementary Information:**

The online version contains supplementary material available at 10.1186/s12866-022-02602-1.

## Background

Facultative endosymbiotic bacteria are extremely common in insects. Reports suggest that at least 40% of all insects are infected by the facultative endosymbiotic bacterium *Wolbachia* [1, 2], while up to 10% of insect species (and up to 23% of Aranaea species) carry *Spiroplasma,* another facultative endosymbiotic bacterium [3–5]. These two symbionts owe their success to their abilities to affect their host biology and promote their own transmission to the next generation of hosts. One such ability revolves around the manipulation of the hosts reproductive system, this occurs in a diverse range of hosts [6–8], and more specifically in the butterflies *Hypolimnas bolina* for *Wolbachia* [9, 10] and *Danaus chrysippus* for *Spiroplasma* [11]; or play defensive roles against diverse parasites and pathogens of their host, including viruses, other bacteria, or parasitoids [12]. Additionally, both of these maternally inherited symbionts have been suggested to occasionally transfer horizontally between host species [13–16]. Studies have shown that divergent species sharing the same diet [13, 14, 17, 18], or the same parasites [16], are also prone to share similar symbiotic strains. Hybridization between closely related host species may also support such horizontal transfers of the symbionts through the introgressed matriline [15, 19]. Altogether, the diversity of phenotypes associated to these symbionts, and their versatile transmission modes, have made host-symbiont associations excellent study systems for various eco-evolutionary processes. Yet, many host-symbiont interactions remain un-noticed. Thus, we lack a true understanding of the diversity and origin of these symbionts in insects, which in turn challenges the comprehensive study of the evolution of these microbial symbioses.

Most phylogenetic studies on *Wolbachia* and *Spiroplasma* are based on a small set of markers. Such sets often only include some or all of the five Multi Locus Sequence Typing (MLST) markers [20] and the *wsp* gene [21] for *Wolbachia;* and the data is even more restricted for *Spiroplasma*, as no MLST markers are yet available for this symbiont. Although broadly used for symbiont screening, and strain characterization, these markers have been criticized for being highly conserved, and thus for being inadequate for depicting the true strain diversity [22] and evolutionary rate of each symbiont. Instead, recent studies advocate for the use of whole genome data [22, 23], but the production of this genomic data is not without difficulties. Sequencing and assembling the genomes of isolated endosymbionts remain costly and methodologically challenging. This is because it is still not always possible to culture and sequence symbionts in isolation from their hosts [24, 25]. Consequently, although a variety of whole genome sequences are available for both *Wolbachia* and *Spiroplasma*, they are still unlikely representative of the true strain diversity that may exist in nature for these bacteria. Furthermore, by only targeting (I) host species that do not belong to the same natural species communities or environments, and are not (or little) interacting in nature, or (II) species that do not share direct phylogenetic relationships (but see [26]), it will remain difficult for the field to infer any major eco-evolutionary event that may shape symbiosis, including horizontal transfer events of the symbionts between host species. As a multi-strain genome-sequencing project targeting all symbionts of interacting/related host species would represent a considerable financial and time investment, such important genomic material is unlikely to become available in the near future. Until then, other methods that allow the field to accumulate genomic data from a wider diversity of symbiotic strains can be, and have been, considered [27–29].

With the constant development of sequencing techniques, the field of genomic diversity is regularly acquiring new genomic material from a wide array of species [30, 31]. For the order Lepidoptera, we have access to genomic material from most families, and sometimes even over 40 species sequenced per family (e.g. 40 genomes of Nymphalidae, mostly due to the intensive genomic work from the *Heliconius* Genome Consortium [32]. The deposited sequence read archives (SRAs) contain the raw host genomic material, but also reads from various other entities originally considered as ‘trash’ or ‘contaminants’ to many, and often not analyzed nor discussed. This non-target material however offers opportunities for a broad screening of DNA material from diverse hidden endosymbionts, and for building the partial to complete assemblies of various symbiotic microorganisms without having to sequence the symbionts independently of their hosts [27]. Here, we screened for genomic material from *Wolbachia* and *Spiroplasma,* two common insect endosymbiotic bacteria*,* in 1094 SRAs files/samples from 106 unique Lepidopteran species. We described symbiotic infections new to the literature [6] and characterized strain diversity using a phylogenomic rather than a MLST-based phylogenetic approach.

## Results

### Identification of reads originating from the endosymbionts *Wolbachia* and *Spiroplasma*

Using MetaPhlAn2 we identified 58 of the 1094 tested SRAs as being infected with *Wolbachia* (with a threshold > 1000 reads), corresponding to 55 individual samples and 16 species (15.1% of the total 106 species screened here). While only six samples were identified as containing *Spiroplasma* reads, representing five species (4.7%). In comparison, Kraken2 (using our custom databases) identified a larger number of SRAs positive for these same infections. A total of 70 SRAs were identified as containing *Wolbachia,* representing 64 biosamples and 20 host species (19%), with the majority also having reads identified as belonging to the *Wolbachia-*associated phage *WO* (Table 1). While 23 SRAs tested positive for *Spiroplasma,* corresponding to 16 individual samples and nine host species (8.5%). Tables 1 and 2 contain a list of all biosamples and SRAs identified as infected in our screen.

**Table 1:** Summary of the specimens with hits to *Wolbachia* based on Kraken2 and MetaPhlAn2 results

Sample Number	Family	Species	Biosample	SRA Accession	Kraken2 (Number of reads)		MetaPhlAn 2 (Number of reads)
					***Wolbachia***	Phage ***Wo***	
1	Adelidae	*Adela reaumurella* (Linnaeus, 1758)	SAMN08536812	SRR6727426	7705	1111	163,166
2	Depressariidae	*Depressaria pastinacella*^*a*^ (Goeze, 1783)	SAMN08712583	SRR6984048 / SRR6984049 / SRR6984052 / SRR6984053 /SRR6984054	1,794,372	75,297	934,134
3	Erebidae	*Hyphantria cunea* (Drury, 1773)	SAMN10290292	SRR8109452	5271	243	–
4	Erebidae	*Hyphantria cunea* (Drury, 1773)	SAMN10290285	SRR8109455	10,719	395	–
5	Gelechiidae	*Keiferia lycopersicella* (Walsingham, 1897)	SAMN10666979	SRR8386696	14,436	533	42,874
6	Gelechiidae	*Keiferia lycopersicella* (Walsingham, 1897)	SAMN10666981	SRR8386698	23,545	822	70,593
7	Gelechiidae	*Keiferia lycopersicella* (Walsingham, 1897)	SAMN10666980	SRR8386699	25,589	964	75,737
8	Gelechiidae	*Keiferia lycopersicella* (Walsingham, 1897)	SAMN10666976	SRR8386700	26,240	882	74,626
9	Gelechiidae	*Keiferia lycopersicella* (Walsingham, 1897)	SAMN10666977	SRR8386701	5676	218	15,557
10	Gelechiidae	*Keiferia lycopersicella* (Walsingham, 1897)	SAMN10666974	SRR8386708	15,323	420	42,046
11	Gelechiidae	*Keiferia lycopersicella* (Walsingham, 1897)	SAMN10666975	SRR8386709	13,604	353	37,698
12	Gelechiidae	*Phthorimaea operculella* (Zeller, 1873)	SAMN10666968	SRR8386702	58,341	2535	125,480
13	Gelechiidae	*Phthorimaea operculella* (Zeller, 1873)	SAMN10666969	SRR8386703	42,812	1808	90,756
14	Gelechiidae	*Phthorimaea operculella* (Zeller, 1873)	SAMN10666970	SRR8386704	47,813	2201	101,751
15	Gelechiidae	*Phthorimaea operculella* (Zeller, 1873)	SAMN10666971	SRR8386705	47,521	2321	78,887
16	Gelechiidae	*Phthorimaea operculella* (Zeller, 1873)	SAMN10666972	SRR8386706	42,526	1925	83,015
17	Gelechiidae	*Phthorimaea operculella* (Zeller, 1873)	SAMN10666967	SRR8386711	37,194	1894	78,586
18	Gelechiidae	*Tuta absoluta* (Meyrick, 1917)	SAMN10666962	SRR8386712	20,483	948	56,469
19	Gelechiidae	*Tuta absoluta* (Meyrick, 1917)	SAMN10666963	SRR8386713	47,837	2141	133,451
20	Gelechiidae	*Tuta absoluta* (Meyrick, 1917)	SAMN10666964	SRR8386714	51,727	2274	146,211
21	Gelechiidae	*Tuta absoluta* (Meyrick, 1917)	SAMN10666965	SRR8386715	53,957	2340	156,218
22	Gelechiidae	*Tuta absoluta* (Meyrick, 1917)	SAMN10666958	SRR8386716	50,877	2202	145,524
23	Gelechiidae	*Tuta absoluta* (Meyrick, 1917)	SAMN10666959	SRR8386717	56,249	2364	160,054
24	Gelechiidae	*Tuta absoluta* (Meyrick, 1917)	SAMN10666960	SRR8386718	90,669	3945	263,050
25	Gelechiidae	*Tuta absoluta* (Meyrick, 1917)	SAMN10666961	SRR8386719	42,398	1860	117,505
26	Geometridae	*Operophtera brumata*^*a*^ (Linnaeus, 1758)	SAMN03121611	SRR1618545 / SRR1618582 / SRR1618581	490,973	18,035	1,171,280
27	Gracillariidae	*Cameraria ohridella* (Deschka & Bimic, 1986)	SAMN07172872	SRR5626452	1289	-	1543
28	Hesperiidae	*Udranomia orcinus* (Felder & Felder, 1867)	SAMN06232397	SRR7174560	11,407	52	10,690
29	Micropterigidae	*Micropterix facetella* (Zeller, 1851)	SAMN08536841	SRR6727435	391,265	1745	263,633
30	Noctuidae	*Chrysodeixis includens* (Walker, 1858)	SAMN06835216	SRR5754050	1287	35	–
31	Noctuidae	*Spodoptera litura* (Fabricius, 1775)	SAMN06187070	SRR5132392	3605	135	1500
32	Noctuidae	*Spodoptera litura* (Fabricius, 1775)	SAMN06187034	SRR5132393	1663	62	2057
33	Noctuidae	*Spodoptera litura* (Fabricius, 1775)	SAMN06187071	SRR5132396	2401	84	1428
34	Noctuidae	*Spodoptera litura* (Fabricius, 1775)	SAMN06187040	SRR5132402	5285	194	2935
35	Noctuidae	*Spodoptera litura* (Fabricius, 1775)	SAMN06187037	SRR5132403	1849	68	1287
36	Noctuidae	*Spodoptera litura* (Fabricius, 1775)	SAMN06187033	SRR5132404	229,970	1672	803,943
37	Noctuidae	*Spodoptera litura* (Fabricius, 1775)	SAMN06187043	SRR5132409	1773	65	-
38	Noctuidae	*Spodoptera litura* (Fabricius, 1775)	SAMN06187073	SRR5132419	1152	18	-
39	Noctuidae	*Spodoptera litura* (Fabricius, 1775)	SAMN06187030	SRR5132426	1678	54	1322
40	Noctuidae	*Spodoptera litura* (Fabricius, 1775)	SAMN06187076	SRR5132427	2383	79	-
41	Noctuidae	*Spodoptera litura* (Fabricius, 1775)	SAMN06187047	SRR5132432	2271	76	-
42	Noctuidae	*Spodoptera litura* (Fabricius, 1775)	SAMN06187032	SRR5132433	170,164	1114	617,094
43	Noctuidae	*Spodoptera litura* (Fabricius, 1775)	SAMN06187038	SRR5132437	6641	201	2529
44	Noctuidae	*Spodoptera litura* (Fabricius, 1775)	SAMN06187072	SRR5132442	395	141	1356
45	Nymphalidae	*Heliconius erato demophoon* (Ménétriés, 1855)	SAMN05224183	SRR4032094	1688	13	1442
46	Nymphalidae	*Heliconius erato demophoon (*Ménétriés, 1855)	SAMN08278546	SRR6432897	1,259,318	21,957	–
47	Nymphalidae	*Hypolimnas misippus* (Linnaeus, 1764)	SAMN10740678	SRR8549338	-	-	1152
48	Nymphalidae	*Pararge aegeria* (Linnaeus, 1758)	SAMN02688782	SRR1190479	33,302	323	1,261,640
49	Nymphalidae	*Pararge aegeria* (Linneaus, 1758)	SAMN09760079	SRR7637637	27,561	310	1,033,664
50	Nymphalidae	*Pararge aegeria* (Linneaus, 1758)	SAMN09760078	SRR7637638	29,222	255	1,245,913
51	Nymphalidae	*Polygonia c-album* (Linneaus, 1758)	SAMN02688783	SRR1190476	146,109	1682	452,747
52	Papilionidae	*Parnassius apollo* (Linneaus, 1758)	SAMN08456343	SRR6679361	6463	-	–
53	Plutellidae	*Plutella australiana* (Landry & Hebert, 2013)	SAMN07626876	SRR6023624	254,213	2402	-
54	Plutellidae	*Plutella australiana* (Landry & Hebert, 2013)	SAMN08388765	SRR6505268	210,829	2090	661,700
55	Plutellidae	*Plutella australiana* (Landry & Hebert, 2013)	SAMN08388772	SRR6505269	318,891	2974	1,032,126
56	Plutellidae	*Plutella australiana* (Landry & Hebert, 2013)	SAMN08388766	SRR6505270	53,109	269	149,256
57	Plutellidae	*Plutella australiana* (Landry & Hebert, 2013)	SAMN08388767	SRR6505271	26,201	288	62,939
58	Plutellidae	*Plutella australiana* (Landry & Hebert, 2013)	SAMN08388768	SRR6505272	61,027	327	171,131
59	Plutellidae	*Plutella australiana* (Landry & Hebert, 2013)	SAMN08388762	SRR6505273	156,370	1532	386,427
60	Plutellidae	*Plutella australiana* (Landry & Hebert, 2013)	SAMN08388769	SRR6505274	84,147	825	207,246
61	Plutellidae	*Plutella Australiana* (Landry & Hebert, 2013)	SAMN08388764	SRR6505276	195,426	1155	524,931
62	Plutellidae	*Plutella Australiana* (Landry & Hebert, 2013)	SAMN08388771	SRR6505279	248,327	1429	870,067
63	Plutellidae	*Plutella australiana* ^*a*^ (Landry & Hebert, 2013)	SAMN08388770	SRR6505277	238,792	2363	1,146,122
64	Plutellidae	*Plutella xylostella* (Linneaus, 1758)	SAMN08388733	SRR6505226	-	-	1727
65	Plutellidae	*Plutella xylostella* (Linneaus, 1758)	SAMN08388732	SRR6505227	2,345,019	9539	-
66	Tineidae	*Tineola bisselliella* (Hummel, 1823)	SAMN08536677	SRR6727411	1867	46	-

^a^represent specimens that had more than one positive SRA accession, for these samples the numbers represented are across all SRA runs

**Table 2:** Summary of the specimens with hits to *Spiroplasma* based on screening with Kraken2 and MetaPhlAn2

Sample Number	Host Family	Host Species	Biosample	SRA Accession	Kraken2 (Number of reads)	MetaPhlAn2 (Number of reads)	Spiroplasma Clade
130	Lycaenidae	*Jalmenus evagoras* (Donovan, 1805)	SAMN08456344	SRR6679362	1152	-	NA
131	Nymphalidae	*Danaus plexippus*	SAMN02986443	SRR1549529	4014	–	III
132	Nymphalidae	*Danaus plexippus* (Linneaus, 1758)	SAMN02986460	SRR1552228	37,369	25,020	II
133	Nymphalidae	*Danaus chrysippus* (Linneaus, 1758)	SAMN02996390	SRR1552518	1147		III
134	Nymphalidae	*Heliconius ethilla narcaea* (Godart, 1819)	SAMN04410681	SRR3103847	22,556	–	I
135	Nymphalidae	*Heliconius congener* (Weymer, 1890)	SAMN04412542	SRR3102172	9891	3171	I
136	Nymphalidae	*Heliconius erato petiverana* (Doubleday, 1847)	SAMN05224118	SRR4032023	1054	–	I
137	Nymphalidae	*Heliconius erato hydara* (Hewitson, 1867)	SAMN05224153	SRR4032061	2840	–	I
138	Nymphalidae	*Heliconius erato erato* (Linnaeus, 1758)	SAMN05224160	SRR4032069	7202	13,008	I
139	Nymphalidae	*Heliconius erato phyllis* (Fabricius, 1775)	SAMN05224205	SRR4032004	13,243	4904	I
140	Nymphalidae	*Heliconius erato emma* (Riffarth, 1901)	SAMN08049958	SRR6313533	11,058	–	III
141	Nymphalidae	*Heliconius erato favorinus* (Hopffer, 1874)	SAMN08049959	SRR6313540	1005	–	NA
142	Nymphalidae	*Danaus chrysippus*^*a*^ (Linneaus, 1758)	SAMN08826815	SRR6925894/ SRR6925895/ SRR6925896/ SRR6925897/ SRR6925899/ SRR6679360	36,983	-	III
143	Nymphalidae	*Heliconius ismenius* (Latreille, 1817)	SAMN09206389	SRR7162650	7817	2425	III
144	Nymphalidae	*Heliconius hecale* (Fabricius, 1776)	SAMN09206391	SRR7162652	43,795	13,919	I
145	Saturniidae	*Antheraea yamamai*^*a*^ (Guérin-Méneville, 1861)	SAMN06758611	SRR5641446/ SRR5641447/ SRR5641448	6559	–	NA

^a^represent specimens that had more than one positive SRA accession, for these samples the numbers represented are across all SRA runs

Neither software identified any sample as being infected by both bacterial symbionts simultaneously. Generally speaking, the majority of samples identified in the MetaPhlAn2 analysis were also identified using Kraken2 (with the exception of two SRAs; Fig. S1). The *Wolbachia* analysis with MetaPhlAn2 identified two SRAs that were not positive in the Kraken2 analysis (Sample number 47 - SRR8549338 and number 64 - SRR6505226). A closer look at these two samples showed that while MetaPhlAn2 identified 1152 and 1727 reads as *Spiroplasma*, Kraken2 only identified 60 and 620 for each sample, respectively and showed no presence of the *Wolbachia*-associated phage *WO.* Due to the small number of Kraken2 identified reads, and phage absence (whose presence has been associated with strong support for *Wolbachia* infection [28]) these samples were not taken forward for further analysis, and are likely to represent false positives.

Overall, the incidence levels for both symbionts in our dataset are slightly lower but comparable to those suggested by the available literature. *Wolbachia* infects 40–80% of all arthropod species [1, 33, 34], and *Spiroplasma* is expected in only 5–30% of all terrestrial arthropod species [5, 35]. Nine of the Lepidoptera species positive here for *Wolbachia* or *Spiroplasma* infection were previously reported to carry the infections in a comprehensive review on symbiont infection in Lepidoptera by Duplouy & Hornett [6]. *Wolbachia* was indeed previously reported in eight of these species, including *Heliconius erato* (however subspecies *H. erato chesstertonii* only [36], which is not included in our sample), *Pararge aegeria* [5], *Parnassius apollo* [37], *Polygonia c-album* [38], *Operophtera brumata* [39], *Plutella australiana* [40], *P. xylostella* [41, 42], and *Tuta absoluta* [43]; while *Spiroplasma* was already previously detected, and intensively studied, in the African monarch *D. chrysippus* [11, 44]. These results suggest that most of the host-symbiont associations detected here have yet to be described and studied in their natural habitats.

### Identification of potential contamination and parasitoids

As we are screening pre-existing genomic data from a variety of tissue types, ranging from specific tissues to whole bodies, the possibility exists that the *Wolbachia* and *Spiroplasma* infections might be those of parasites, parasitoids or host plants of the Lepidoptera, as opposed to the targeted *Lepidoptera* species themselves. A complete summary of contaminant groups is given in File S1. Within, the samples positive for *Spiroplasma*, two (Sample Numbers: 140, 131) had 0.1% of the total reads assigned to Hymenoptera, and a further three (Sample Numbers: 130, 136, 145) were positive for the plant phyla Streptophyta, with Sample Number 145 having ~ 10% of reads assigned to a potential host plant, *Vigna unguiculata*. In comparison, of the 64 biosamples identified as positive for *Wolbachia*, two (Sample Numbers: 2, 30) had 0.1% of reads assigned to Hymenoptera or 0.3% to Coleoptera, respectively (See Table 3). A further three samples (Sample Numbers: 2, 37, 45) were positive for the plant phyla Stretophyta, with up to 1% of reads being assigned to the phylum.

**Table 3:** Summary of samples for which more than 0.1% of overall reads were assigned to possible contaminant taxa

Sample Number	Accession	Species	Positive for	Tissue Type	Percentage of reads assigned to group
2	SAMN08712583	*Depressaria pastinacella*	*Wolbachia*	Whole Insect	0.1% - Hymenoptera 0.87% - Streptophyta
30	SRR5754050	*Chrysodeixis includens*	*Wolbachia*	Whole body	0.3% - Coleoptera
37	SRR5132409	*Spodoptera litura*	*Wolbachia*	Whole body	0.21% - Streptophyta
45	SRR4032094	*Heliconius erato demophoon*	*Wolbachia*	Whole body	0.1% - Streptophyta
130	SRR6679362	*Jalmenus evagoras*	*Spiroplasma*	Thorax	0.2% - Streptophyta
131	SRR1549529	*Danaus plexippus*	*Spiroplasma*	Thoracic muscle	0.1% - Hymenoptera
136	SRR3102172	*Heliconius erato petiverana*	*Spiroplasma*	Whole body	0.1% - Streptophyta
140	SAMN06758611	*Heliconius erato emma*	*Spiroplasma*	Whole body	0.1% - Hymenoptera
145	SAMN06758611	*Antheraea yamamai*	*Spiroplasma*	Whole body	10% - Streptophyta

### *Wolbachia*

Two to three rounds of baiting of *Wolbachia* originating reads with mirabait revealed that on average 6.35% of the total number of reads from each *Wolbachia*-infected SRA are of the symbiont (range: 0.002–54.26%, File S2). The resulting extracted reads were used to construct 64 partial assemblies, with 18 being < 0.5 Mbp in size. On average, the assemblies consisted of a total size of 0.9 Mbp spread across 245 contigs. Complete assembly statistics can be found in File S2. Identification of BUSCO genes found on average 87 Complete and Single copy orthologues (Range: 0–131, File S2), in comparison 121 genes (out of 148 total) were identified from the reference genomes (File S3). The samples that lacked the identification of any BUSCO genes were those of < 0.1 Mbp in size, which is expected due to their incomplete and fragmented nature. Noticeably, 12 of the assemblies lacking gene identification belonged to *Spodoptera litura*. Following manual curation of the dataset a total of 69 *Wolbachia* strains (21 references and 48 samples) and 133 genes were included in the phylogenetic analysis. Concatenation of the BUSCO genes sequences resulted in a final alignment of 141,346 bp.

The MLST and *wsp* genes were identified for 68 strains (47 samples and 21 references), with concatenation resulting in a final alignment of 2539 bp. In addition to the concatenated alignment, alignments for each of the five MLST and one *wsp* genes were carried forward. All five MLST genes were identified in 48/68 (36 samples and 12 references) strains, while the *wsp* gene was present in 15/68 (5 samples and 10 references) strains.

**Fig. 1:**
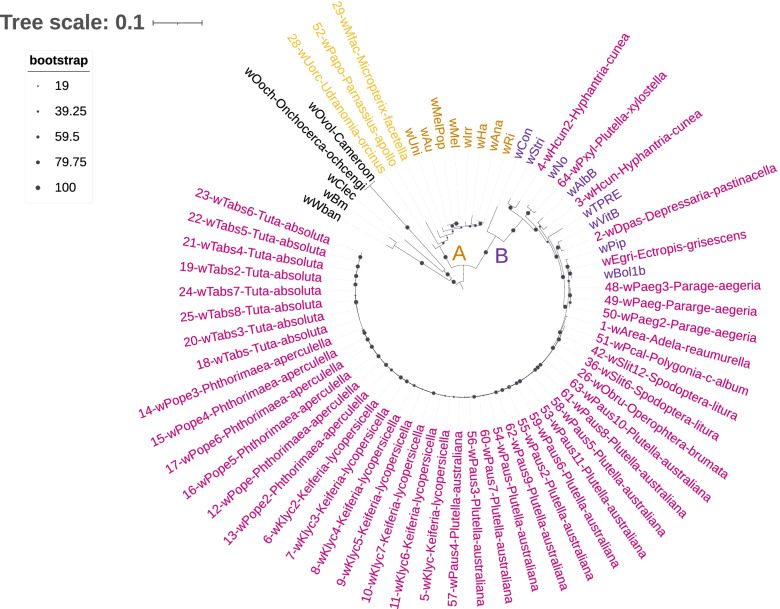
Phylogenetic relationships between *Wolbachia* positive SRAs and reference genomes based on 133 BUSCO genes. Tree was rooted using the reference genomes of the C- D- and F-supergroups (black, *N* = 5 reference genomes). All samples characterized in this study (annotated with a sample number and host species) belong to either the A-supergroup (orange, *N* = 3 + 8 reference genomes) or the B-supergroup (purple, *N* = 45 + 8 reference genomes)

**Fig. 2:**
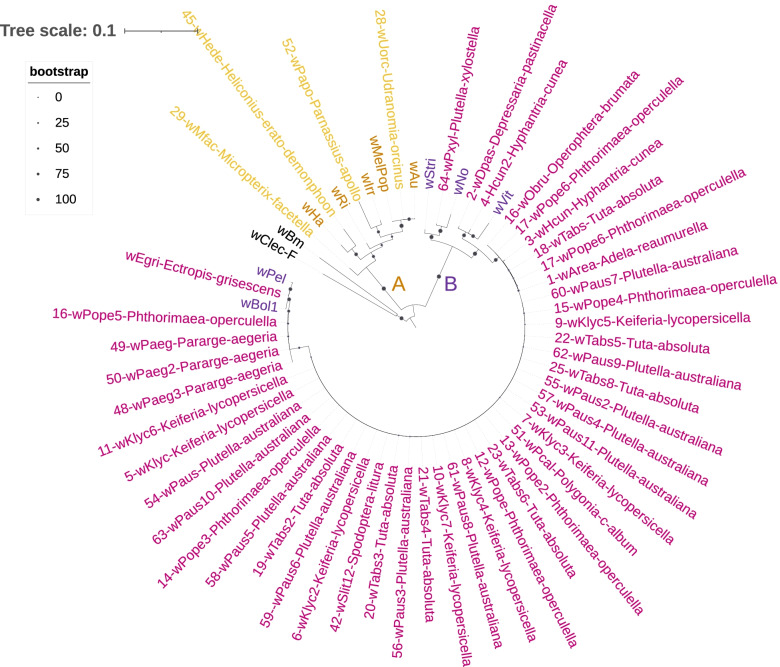
Phylogenetic relationships between *Wolbachia* positive SRAs and reference genomes based on five MLST and the wsp genes. Tree was rooted using reference genomes data from *Wolbachia* strains *w*Bm and *w*Clec-F from the D- and F-supergroup respectively (in black). All samples characterized here (annotated with a sample number and host species) belong to either the A-supergroup (orange, *N* = 4 + 5 reference strains) or the B-supergroup (purple, *N* = 44 + 5 reference strains)

All the *Wolbachia* strains identified from our samples belong to the A- and B- supergroups; with the majority (44/48, 92%) belonging to the B-supergroup. Despite using a slightly different set of strains, similar tree configurations were obtained by using the concatenated BUSCO sequences or the concatenated sequences of the MLSTs and *wsp* genes (Figs. 1 and 2). Finally, the phylogenies based on only one single MLST gene or the *wsp* gene showed the same groupings of samples into either A- and B- supergroups, with generally fewer representative samples per phylogeny, and lower resolution among individual samples (Figs. S2, S3, S4, S5, S6 and S7.

**Fig. 3:**
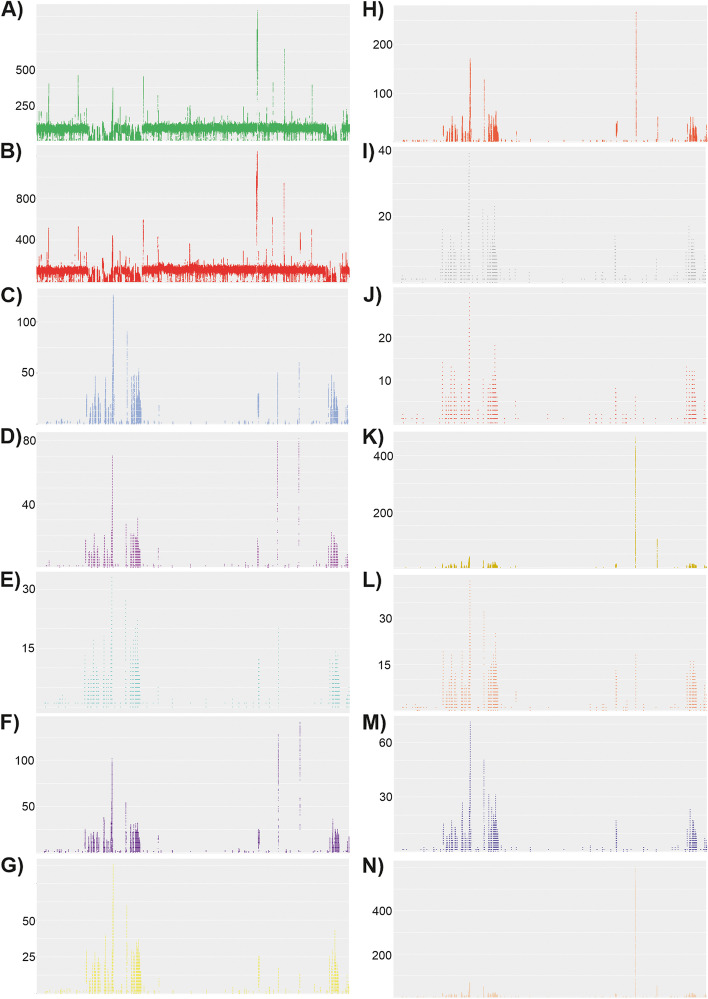
Mapping of the 14 *Wolbachia* positive *Spodoptera litura* samples along the *w*Pip genome. Reads identified as belonging to *Wolbachia* were mapped to the *w*Pip *Wolbachia* reference genome (GCF_000073005.1). Coverage values shown on the vertical side of the figure. The graphs correspond to the following samples, (represented as sample number, as given in Table 1, − SRA Accession): **A**) 42 - SRR5132433; **B**) 36 - SRR5132404; **C**) 34 - SRR5132402; **D**) 33 - SRR5132396; **E**) 32 - SRR5132393; **F**) 31 - SRR5132392; **G**) 44 - SRR5132442; **H**) 43 - SRR5132437; **I**) 39 - SRR5132426; **J**) 38 - SRR5132419; **K**) 37 - SRR5132409; **L**) 35 - SRR5132403; **M**) 40 - SRR5132427; **N**) 41 - SRR5132432

Interestingly, 14 SRAs from the unique bioproject: PRJNA344815 [45] were identified as being positive for *Wolbachia* under our criteria. However, only two produced relatively complete assemblies (> 1.2 Mbp in length) from which BUSCO and MLST genes could be extracted. To determine whether the remaining 12 SRAs potentially represented false positives or potentially host insertion of *Wolbachia* gene(s) within the host genome a mapping approach was taken. The mapping of the reads baited out during the second round of baiting with mirabait against the *w*Pip genome (GCF_000073005.1) show for the two samples that produced good assemblies (Sample Number 42 - SRR5132433 and Number 36 - SRR5132404) high levels of coverage is seen along the entire *w*Pip genome (Fig. 3A and B). In comparison, the remaining 12 SRAs showed low and sometimes patchy coverage along the reference genome (Fig. 3C - N). However, due to the distribution of reads along the reference genome these samples are thought to represent true infection, with low numbers of sequencing reads originating from *Wolbachia*, resulting in overall low coverage and hence the inability to generate reasonable assemblies for gene extraction and phylogenetic analysis. Nevertheless, this highlights that although it might not be possible to always assemble a symbiont genome from positive samples, the screening approach used here provides interesting testable hypotheses for further work.

### *Spiroplasma*

Baiting of *Spiroplasma* reads identified that on average 2.38% of the total number of reads in infected SRA files belonged to the symbiont (Range: 0.0004–19.75%, File S4). A maximum of two baiting rounds was required for optimal sequence baiting of *Spiroplasma*. Of the 16 samples identified as being positive, only 15 produced assemblies. In the case of SAMN06758611 (Sample Number 145) none of the produced contigs blasted to any of the *Spiroplasma* references, and therefore this sample was discarded from further analysis.

Identification of BUSCO genes found on average 27 single copy complete orthologs (Range 0–47, File S4), in comparison 55 genes (File S3) were extracted from the reference genomes (148 BUSCO genes total). The samples that lacked identification of any BUSCO genes were those with assemblies of < 0.1 Mbp in size. Following manual curation of the dataset, a total of 55 *Spiroplasma* strains (42 references + 13 samples) and 63 genes were used for phylogenetic analysis. Concatenation resulted in a final alignment of 40,401 bp.

**Fig. 4:**
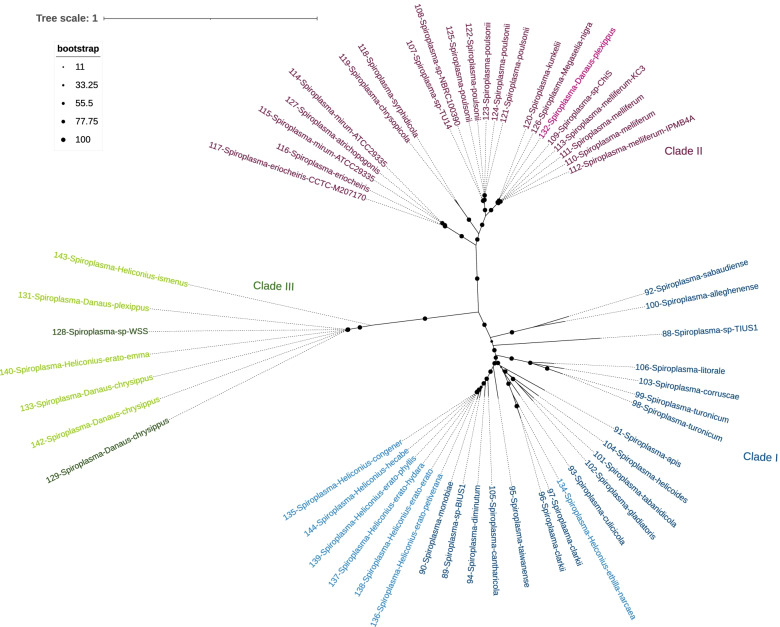
Phylogenetic relationship between *Spiroplasma* positive SRAs and reference genomes based on 63 BUSCO genes. All samples characterized here are annotated with a sample number, followed by either the host species (for SRA screened samples) or the *Spiroplasma* species (for reference samples) belong to either the Clade I (blue), II (red) or III (green). All reference samples are coloured with a darker shade of the Clade colour

Based on the BUSCO phylogeny, all the *Spiroplasma* strains identified from our samples are similar to previously characterized strains (ie. reference genomes) (Fig. 4); *Spiroplasma* is here distributed among three clades: Clade I, or the *Apis* clade as described by Gasparich et al [46], contains most of the *Spiroplasma-*infected SRAs (7 in total), all of which group together with *Spiroplasma* strains originating from Hymenoptera, Diptera and Coleoptera hosts, including *Spiroplasma apis, S. clarkia* and *S. sabaudiense*; Clade II, or the Citri-Chrysopirola-Mirum clade [46], includes only a single SRA sample, which is grouped with *Spiroplasma* reference genomes from *Diptera* and *Hymenoptera* hosts (ie. *S. mirum, S. chrysopirola* and *S. poulsonii*); lastly, Clade III consists of five SRA samples grouped with the single Lepidoptera *Spiroplasma* reference (from *Danus chrysippus*), and a Hymenoptera reference. It remains unknown whether Clade III corresponds to the ixodetis clade described by Gasparich et al [46], as the species included in the ixodetis clade and our Clade III were reciprocally absent between the two studies.

## Discussion

By screening 1094 SRAs files from diverse Lepidoptera species for genomic material from *Wolbachia* and *Spiroplasma* symbionts, we isolated and characterized infections by either of these two symbiotic bacteria in 28 Lepidoptera species. For many of these host species, these infections had, to our knowledge, never been described [6]. Noticeably, some of these newly discovered infections were found in species with strong prior ecological and evolutionary knowledge. In particular, our screening work revealed an additional five *Heliconius* species (including six subspecies of *H. erato*) infected with *Spiroplasma*, and one species with a *Wolbachia* infection. Previous studies had identified *Spiroplasma* from *H. clysonymus* [47], *H. doris* [48], and *H. aoedes* [49], while *Wolbachia* was identified from *H. cydno* [47] and *H. erato chestertonii* and *H. e. venus* [36]. Additionally, the taxonomy browser in NCBI suggests an additional 16 species and subspecies of *Heliconius* carrying *Spiroplasma* (*H. charithonia, H. clysonymus, H. cydno chioneus, H. demeter, H. e. notabilis, H. eratosignis, H. melpomene, H. m. amaryllis, H. m. meriana, H. pachinus, H. sara, H. telesiphe, H. timareta, H. t. timareta, H. wallacei, and H. xanthocles*). Altogether, these results suggest that endosymbiotic bacteria commonly infect the *Heliconius* butterfly clade. What however remains surprising is that despite more than 350 studies (Pubmed Dec 2021) using *Heliconius* butterflies as study organisms, one has yet to experimentally test the role of these symbionts in these species. With *Spiroplasma* and *Wolbachia* infecting *Heliconius* species from different clades across the *Heliconius* phylogeny [50], one could for example wonder whether these symbionts have played a role in the speciation and diversification of this species rich insect genus.

Despite our efforts to optimize the detection of symbiotic infections and the extraction of their genomic material from the SRA samples (not specifically aimed at metagenomics analysis), we could only produce the partial genomic assemblies of 64 *Wolbachia* strains and 15 *Spiroplasma* strains. Three hypotheses can explain the incompleteness of our assemblies: (a) low sequencing data quality and/or incomplete sequencing of the entire symbionts genomic chromosomes; (b) contamination with DNA from another samples; (c) insertion of some genomic material from the symbiont(s) within the genome of the host. Methodological boundaries to the detection and construction of full symbiotic genomes are several folds. The use of Kraken2 for screening the SRAs yields more positive results than the screening using the default MetaPhlAn2, leading to a wider range of genomic assemblies. This is most likely due to the use of a customized reference database with either *Wolbachia* or *Spiroplasma* reference genomes in Kraken2. Nevertheless, both software provided useful results for screening of endosymbionts. Kraken is considered to have good performance metrics in terms of accuracy and abundance profiles [51], but the main advantage to using Kraken2 is the ability to create custom databases, however it requires large amounts of memory (> 100 Gb). In cases where high amounts of memory are unavailable, MetaPhlAn2 is a good alternative and has low computational requirements and fast classification speed [51], however the main limiting flaw is its’ database and the inability to create custom databases, which for us resulted in fewer ‘infected’ samples.

Kraken2 is also likely to produce some false negative results, simply because the SRA data we screened was not optimized for the sequencing and analyses of metagenomes. For example, the tissue type used for extraction, is likely to affect if or how much endosymbiotic bacteria ends up in the final DNA library. An arbitrary cut off of 1000 reads was chosen as we considered fewer reads than this would result in insufficient data to yield a useful assembly result, therefore samples with slightly lower than 1000 *Wolbachia* or *Spiroplasma* reads could also potentially represent ‘true’ positives that may warrant further investigation. Our protocol was optimized to make sure each step significantly improved the quality of our final assemblies in a time-efficient manner.

Additionally, the endosymbiont genomes we constructed might potentially have originated from contaminant parasites, or associated host plant, as opposed to the targeted Lepidoptera. Since the data investigated in this study has been obtained from publically available genomic data, we have no control over the tissue types used for extraction, or the sample preparation. To investigate potential contamination, we took a conservative approach and listed all samples for which > 0.1% of the reads belonged to potential ‘contaminant’ taxa (ie. parasitoid, parasite, or host plant). Although it should be noted that there is no consensus on which thresholds should be used with metagenomic taxonomic classifiers [51, 52], with thresholds being considered on a project specific basis, we believe that the endosymbiont genomes presented in this study originate from the Lepidoptera host targeted by the sequencing project, with possibly one notable exception. The sample number 145 includes 10% of reads assigned to the plant *Vigna unguiculata*. However, the assembly produced from the baited *Spiroplasma* reads from this sample was discarded from downstream analysis, due to none of the assembled contigs blasting to any *Sprioplasma* assemblies from our reference database. Nonetheless, the true infection status of many of the species screened for infection here remains to be confirmed through screening of fresh wild samples. Similarly, the true role of these infections will only be fully tested through ecological studies in the hosts respective natural habitats.

Despite the shortcomings, the 48 *Wolbachia* and 13 *Spiroplasma* partial genomic assemblies (out of 64 and 15 produced in this study, respectively) included enough target genes to support phylogenomic analyses of the symbiotic strains. The *Wolbachia* phylogenetic trees built either using the MLST sequences only, or using the BUSCO genes sequences, both similarly divided the strains within the A- and B-supergroups, with a higher number of strains belonging to the B-supergroup. This observation is not new, as Lepidoptera have been described as hosts to a greater number of B-supergroup *Wolbachia* compared to A-supergroup *Wolbachia* [44, 53–56]. Additionally, the phylogenomic approach using the BUSCO genes revealed higher *Wolbachia* strain diversity than did the MLST-based phylogenetic analyses. This supports the idea that the MLST markers are unsuited for fine-scale strain differentiation in this bacterial clade [22, 23]. Nonetheless, even when using a whole-genome typing method such as the BUSCO genes, the resulting phylogenomic tree highlighted very little divergence within the B-supergroup *Wolbachia* strains, with divergent Lepidoptera species carrying closely related strains. Similarly, although the newly characterized *Spiroplasma* strains belong to three divergent clades, few strains from highly divergent host species show very little genetic differences. Both *Wolbachia* and *Spiroplasma* are vertically transmitted in insects [57, 58], but hybrid introgression and other shared host-resource have been proposed as platforms for the horizontal transfer of these microbial symbionts [14, 58]. As many host species are from different geographic regions and evolved in different environments, it remained impossible to identify which eco-evolutionary routes may have supported the transfer of these symbionts between host species [59].

Additionally, while large, almost complete assemblies of symbiotic chromosome are often evidence for true natural infections, partial symbiont assemblies that are much shorter than 500,000 bp long often require further investigation. As discussed above, we are confident that our quality criteria across our methodology allowed us to remove many potential false positives. In our dataset, however, few SRA samples clearly included genomic material of *Wolbachia* origin, which supported the construction of small assemblies, from which none of the BUSCO or *Wolbachia* MLST genes were retrieved. This was the case for 12 of the 14 positive SRAs from the moth species *Spodoptera litura* (Noctuidae, Fabricius 1775). Closer investigation into these SRAs showed that mapping of the baited reads along the *w*Pip reference genome had low and sometimes patchy coverage. Therefore, we conclude that these are likely to represent true positives, but with insufficient coverage of the *Wolbachia* genome to promote an adequate assembly for gene extraction and phylogenetic analysis, using the approach taken here. Alternative gene identification methods, such as programs designed to identify exons from fragmented genomes [60] could represent an alternative approach to the identification of the corresponding BUSCO genes.

## Conclusions

This study highlights the usefulness of existing genomic data to investigate the true diversity of endosymbiotic bacteria. Here we present two methods to detect the presence of endosymbionts, such as *Wolbachia* and *Spiroplasma.* Generally speaking, provided large amounts of memory are available for computation, Kraken2 identified more samples as containing *Wolbachia* and *Spiroplasma* compared to MetaPhlAn2. We also successfully produced partial endosymbiont genomes that can be mined for phylogenetically informative genes, to better understand their evolutionary histories. The deep analysis of such ‘once hidden symbiont’ genomic material from a wider diversity of hosts and environments, than currently available, will benefit the studies of different eco-evolutionary events associated to the evolution and establishment of bacterial symbioses in arthropods, including radiation, horizontal transfers, and lateral gene transfers.

## Material & methods

### Dataset construction

All samples included in this study are available in the NCBI Sequence Read Archive (SRA). To identify samples for screening, all accession numbers that matched the criteria of Lepidoptera genomic DNA were sent to the NCBI run selector (as of September 2020). Within the run selector, the following criteria were used for sample selection: (i) ran on the illumina platform, (ii) had a WGS assay type, and (iii) paired library layout. This resulted in a total of 1094 samples for analysis, covering 106 species (File S5). We used prefetch and Fasterq-dump v. 2.9.6 from the SRA Toolkit (NCBI SRA) to download the reads from each accession.

### Taxonomic assignment

Reads were assigned taxonomic labels with Kraken2 [61] and MetaPhlAn 2.0 [62]. Kraken2 assigns taxonomic labels using a k-mer based search, whereby each k-mer within a query is matched to the lowest common ancestor of genomes in the database containing the given k-mer, this information is then used by the classification algorithm to infer the taxonomic classification. In comparison, MetaPhlAn2 uses clade-specific marker genes (identified from across ~ 17,000 reference genomes) to determine the taxonomic composition of the input dataset. Kraken2 was run using a confidence threshold of 0.05, a mpa style output and a custom database which contained; (i) the standard kraken database, (ii) Refseq viral database, (iii) Refseq plasmid database, (iv) Refseq bacteria database, (v) Univec core database and (vi) available Lepidoptera sequences (downloaded as of April 2020). A full list of taxa included in the database is given in File S6. MetaPhlAn was run using the analysis type rel_ab_w_read_stats, which provides the relative abundance and an estimate of read numbers originating from each clade. The resulting outputs were screened for lines matching *Wolbachia* or *Spiroplasma*. Based on the Kraken2 results, datasets that contained > 1000 hits to either *Wolbachia* or *Spiroplasma* were taken forward for further analysis. Overlap between each analysis was inferred and Venn diagrams constructed with Jvenn [63].

To rule out possible sources of contamination with material from parasites, parasitoids or host plants, we screened the Kraken2 results for the presence of any Insecta Orders, Arthropod classes, Nematoda, Platyhelminthes and Plant Phyla. Any contaminant groups with > 1000 reads assigned are listed in File S1. The conservative limit of 1000 reads was used as a pre-screen for contaminants. However, due Kraken2 often reporting false positives in relation to low abundance taxa [51, 52], only samples whereby more than 0.1% of the total reads were assigned to ‘contaminants’ are suspected of being contaminated.

### *Wolbachia* and *Spiroplasma* reference database construction

To identify reads originating from either *Wolbachia* or *Spiroplasma* a reference dataset containing genomes from either *Wolbachia* or *Spiroplasma* were constructed. A total of 21 *Wolbachia* and 42 *Spiroplasma* genomes were downloaded from NCBI (September 2020), respectively. For the *Wolbachia* reference database, genomes chosen to represent strains found in the supergroups A, B, C, D and F, with a single representative per stain being included. *Spiroplasma* genomes originating from insect hosts were included in the *Spiroplasma* database. A complete list of the genomes included in each database is shown in File S3.

### Identification and assembly of *Wolbachia* and *Spiroplasma* reads

Independent SRA experiments or runs originating from the same Biosamples that met our screening criteria were combined into a single dataset. To identify reads originating from the endosymbiont, a modified version of Pascar and Chandler [29] method was used. For each sample, reads were extracted from the full dataset that matched at least one kmer to the respective reference dataset using the mirabait tool from MIRA 4.0.2 [64] using a kmer value of 31. The extracted reads were assembled using SPAdes 3.13.1 [65], with the baited reads being considered single end and kmer values of 21, 33 and 55. The resulting contigs were then blasted back to their respective endosymbiont database using standalone blast 2.0.0+ (megablast, evalue threshold of e-10, 70% minimum percentage identity), contigs lacking significant blast hits were removed. The remaining contigs were used as the reference for the second round of baiting, followed by reassembly, and blast search. This process was repeated until a < 5% increase in the number of reads baited was observed, for this dataset no significant increase was seen after three baiting rounds. The assemblies produced at the end of round of either round two or three were carried forward for downstream analysis. All final assemblies are available at Zenodo: 10.5281/zenodo.6517359

### Gene identification, alignment and phylogenetic reconstruction

The following steps were carried out for each symbiont dataset independently. The assemblies resulting from the final round of baiting were used to identify single copy bacterial genes with BUSCO v 3.0.2 [66, 67] in genome mode, utilizing the bacteria odb9 database. Individual genes were aligned based on BUSCO IDs using MAFFT 7.407 [68], using the auto option which chooses the best alignment method based on the data. The resulting gene alignments were manually screened and curated using Geneious Prime® 2020.2.4 (http://www.geneious.com). This step was carried out to ensure correct orthology and alignment. Genes identified in < 10 samples were removed from the final dataset. This resulted in a final dataset of 133 *Wolbachia* and 63 *Spiroplasma* genes.

For all *Wolbachia* assemblies, in addition to the BUSCO genes, the five MLST genes (*CoxA, FbpA, FtsZ, Gatb, HcpA*) and the *wsp* gene were identified using a blast approach and extracted when present. A reference set for each gene was obtained from GenBank (See File S5 for a complete list) with representative strains of the *Wolbachia A*-, B-, F- and D-supergroups. A blast search was carried out against these references using Genious Prime® 2020.2.4, and the corresponding region were extracted from our assemblies. Individual gene alignments were produced using the pairwise Geneious Alignment, default options, in Geneious Prime. Alignments were manually screened to check for correct alignment.

For the phylogenetic analysis of *Spiroplasma* samples, all BUSCO genes were concatenated in alphabetical gene order, resulting in an alignment of 40,401 bp. The following datasets were constructed for the analysis of *Wolbachia* samples: (I) Concatenated BUSCO genes (141,346 bp), (II) Concatenated MLST + *wsp* genes (2539 bp), (III – VIII) Individual MLST and *wsp* genes. Alignments are available from Zenodo doi: 10.5281/zenodo.6517359 All concatenated alignments were double-checked for misaligned regions using AliView [69].

Phylogenetic reconstruction was carried out for each dataset with CIPRES v.3.3 [70] using RAxML-HPC2 on XSEDE [71] with the Gamma+I parameter. Tree visualization and figures were produced with FigTree (http://tree.bio.ed.ac.uk/software/figtree/) and ITOL [72, 73] using the bipartitions output trees produced by RAxML.

### A closer look at the *Spodoptera litura* samples

Since 12 of the 14 SRAs belonging to *Spodoptera litura* produced poor assemblies, for which no BUSCO or MLST genes could be identified, we wanted to further investigate their composition. To determine if the samples potentially represented false positives or host insertions of *Wolbachia* genes rather than true infections a mapping analysis was carried out. Reads baited during the second round of Mirabait were mapped to the *w*Pip reference genome (GCF_000073005.1) with bowtie2 v.2.4.1 [74], using the sensitive local option. The resulting sam files were converted to sorted bam with samtools v1.10 [75]. Coverage information was obtained using samtools depth, and the resulting graphs plotted with the ggplot package [76] in R.

## Supplementary Information

**Additional file 1.**

**Additional file 2.**

**Additional file 3.**

**Additional file 4.**

**Additional file 5.**

**Additional file 6.**

**Additional file 7. Figure S1:** Venn diagram representing the overlap between samples identified as having > 1000 reads associated with either **A**) Wolbachia or **B**) Spiroplasma. MetaPhlan results are represented in Green and Kraken in Blue.

**Additional file 8. Figure S2:** Phylogenetic relationships between Wolbachia positive SRAs and reference sequences for the MLST gene *coxA*.

**Additional file 9. Figure S3:** Phylogenetic relationships between Wolbachia positive SRAs and reference sequences for the MLST gene *fbpa*.

**Additional file 10. Figure S4:** Phylogenetic relationships between Wolbachia positive SRAs and reference sequences for the MLST gene *ftsz*.

**Additional file 11. Figure S5:** Phylogenetic relationships between Wolbachia positive SRAs and reference sequences for the MLST gene *GatB*.

**Additional file 12. Figure S6:** Phylogenetic relationships between Wolbachia positive SRAs and reference sequences for the MLST gene *hcpa*.

**Additional file 13. Figure S7:** Phylogenetic relationships between Wolbachia positive SRAs and reference sequences for the *wsp* gene.

## Data Availability

The raw data analyzed were obtained from the NCBI SRA archive, all SRA numbers are given in File S5. The resulting symbiont assemblies and final gene alignments are available from Zenodo doi: 10.5281/zenodo.6517359
