# Supplementary figures and images for: One’s trash is someone else’s treasure: sequence read archives from Lepidoptera genomes provide material for genome reconstruction of their endosymbionts
Source: BMC Microbiol. 2022 Aug 30;22:209. doi: 10.1186/s12866-022-02602-1 (PMC9426245; doi:10.1186/s12866-022-02602-1)

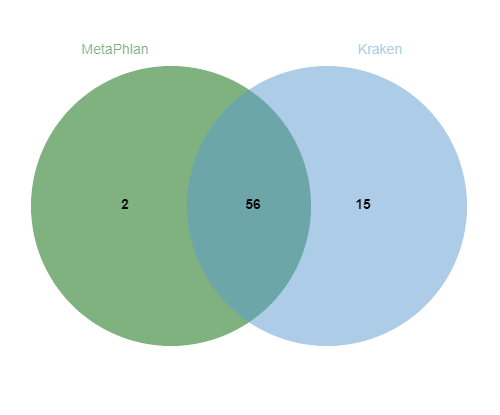

Supplement: Supplementary file 7 — Additional file 7. Figure S1: Venn diagram representing the overlap between samples identified as having > 1000 reads associated with either A) Wolbachia or B) Spiroplasma. MetaPhlan results are represented in Green and Kraken in Blue. [file 12866_2022_2602_MOESM7_ESM.zip › FigS1a_Venn_wolb_ESM.png]

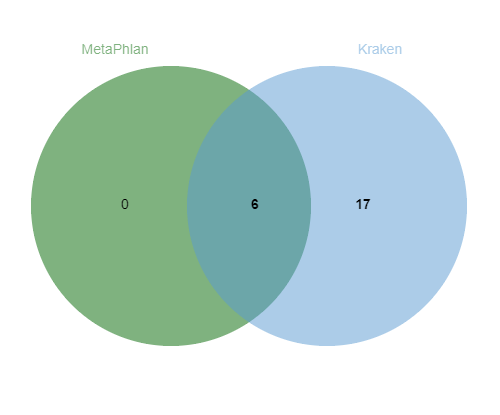

Supplement: Supplementary file 7 — Additional file 7. Figure S1: Venn diagram representing the overlap between samples identified as having > 1000 reads associated with either A) Wolbachia or B) Spiroplasma. MetaPhlan results are represented in Green and Kraken in Blue. [file 12866_2022_2602_MOESM7_ESM.zip › FigS1b_Venn_Spiro_ESM.png]

Tree scale: 0.1

bootstrap

0

25

50

75

100

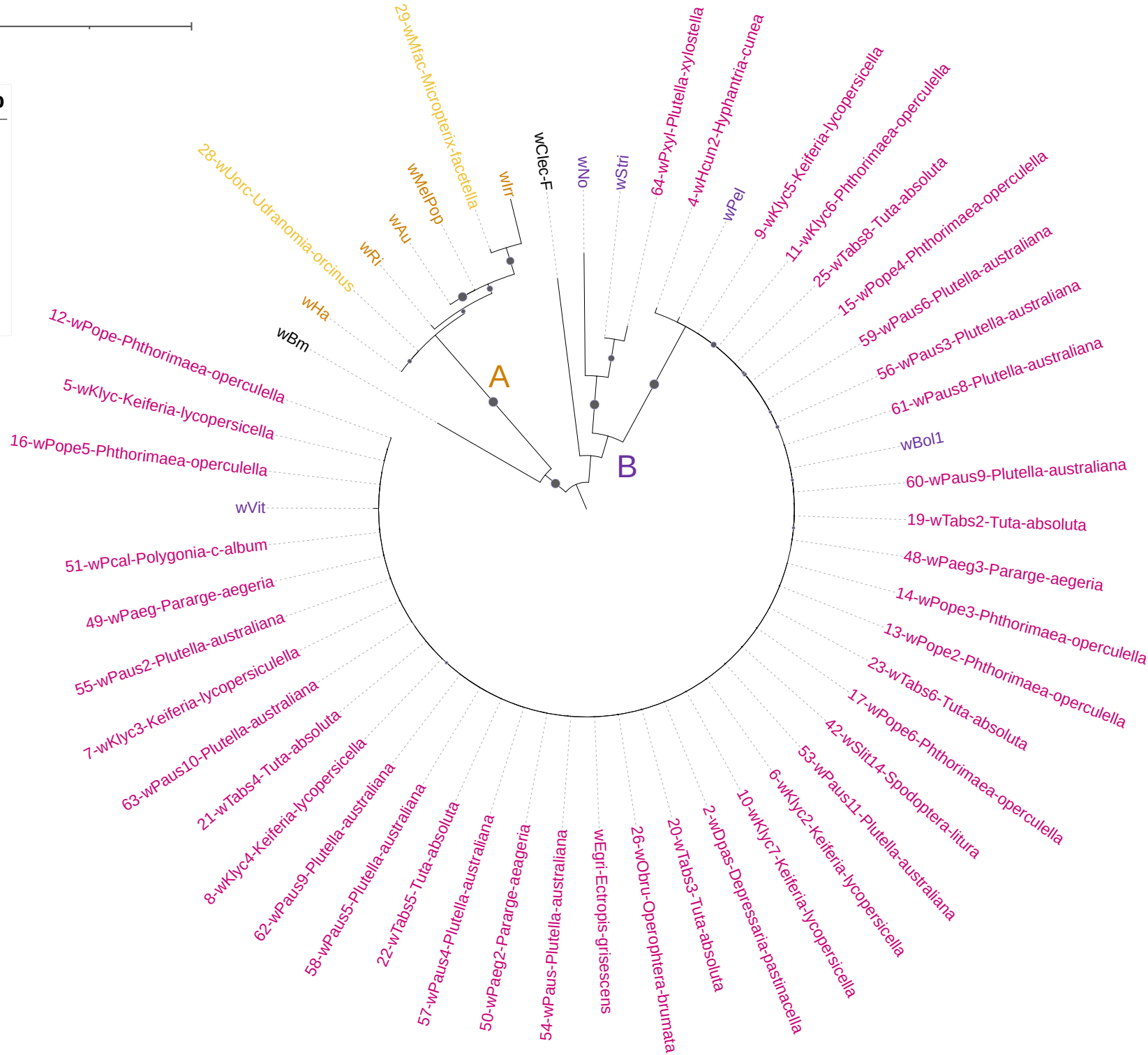

Supplement: Supplementary file 8 — Additional file 8. Figure S2: Phylogenetic relationships between Wolbachia positive SRAs and reference sequences for the MLST gene coxA. [file 12866_2022_2602_MOESM8_ESM.pdf]

Tree scale: 0.1

bootstrap

• 0

• 25

• 50

• 75

• 100

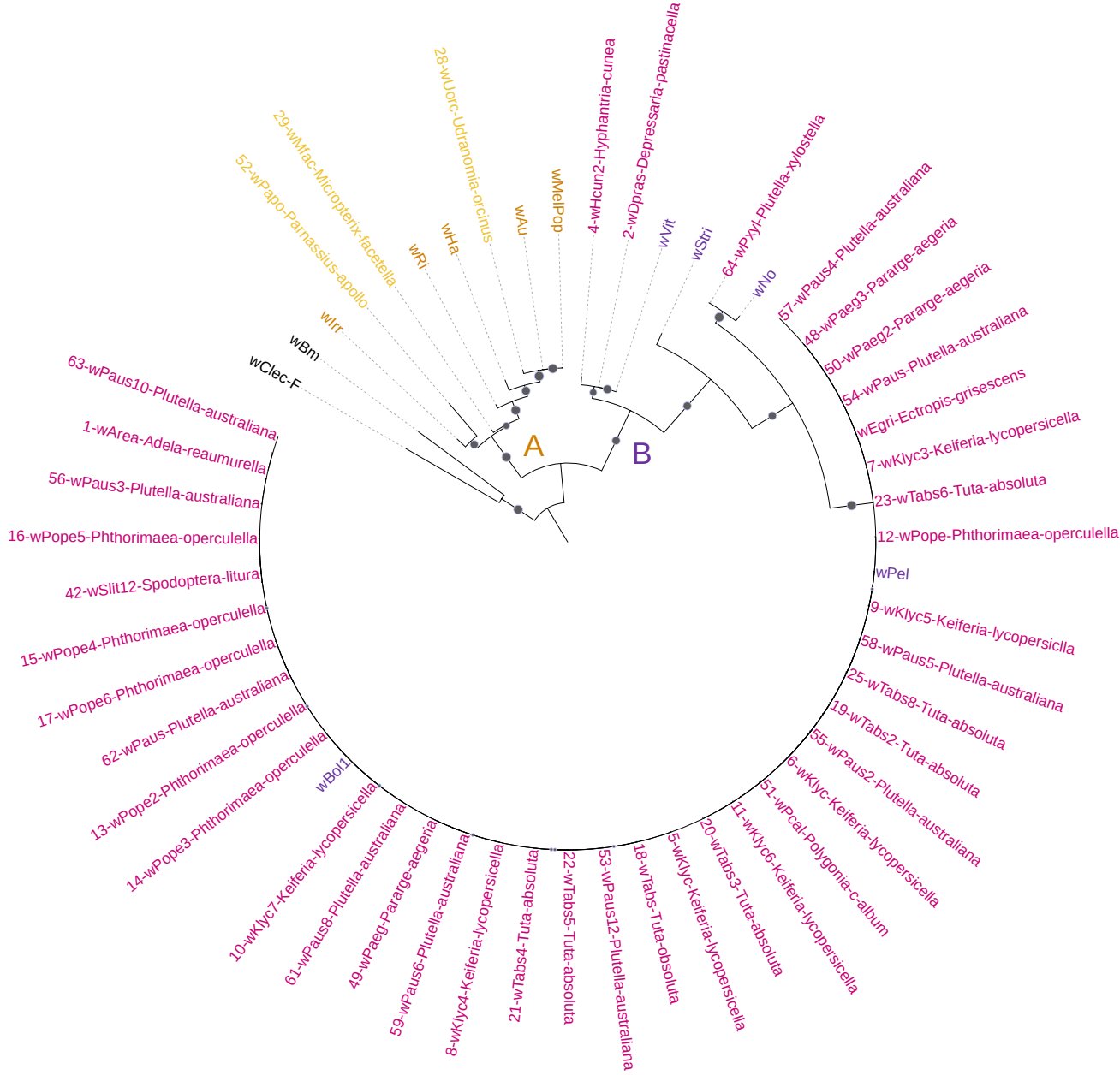

Supplement: Supplementary file 9 — Additional file 9. Figure S3: Phylogenetic relationships between Wolbachia positive SRAs and reference sequences for the MLST gene fbpa. [file 12866_2022_2602_MOESM9_ESM.pdf]

Tree scale: 0.1

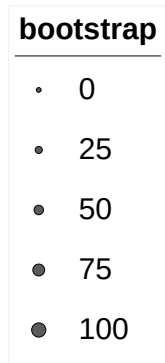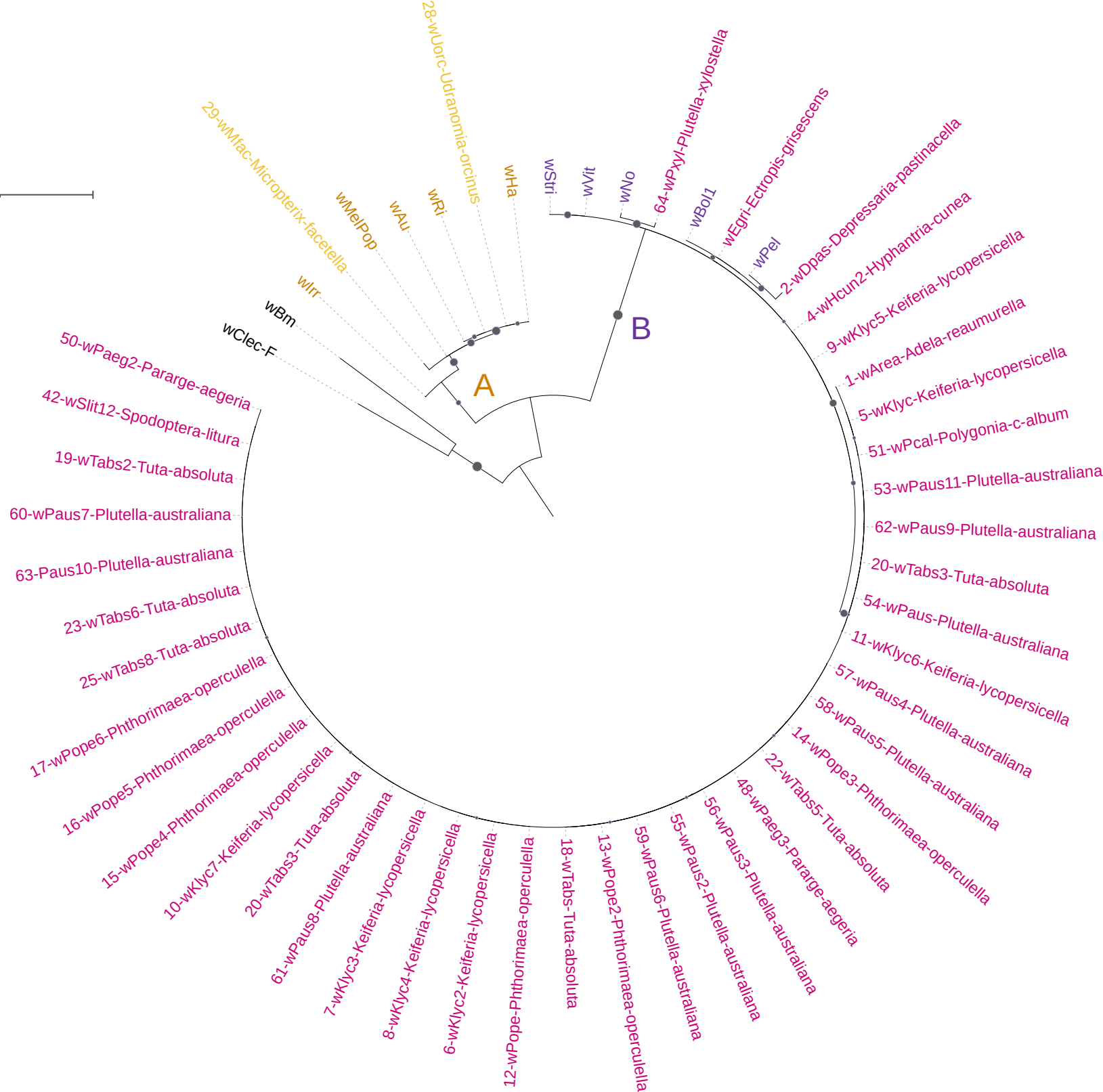

Supplement: Supplementary file 10 — Additional file 10. Figure S4: Phylogenetic relationships between Wolbachia positive SRAs and reference sequences for the MLST gene ftsz. [file 12866_2022_2602_MOESM10_ESM.pdf]

Tree scale: 0.1

bootstrap

0

25

50

75

100

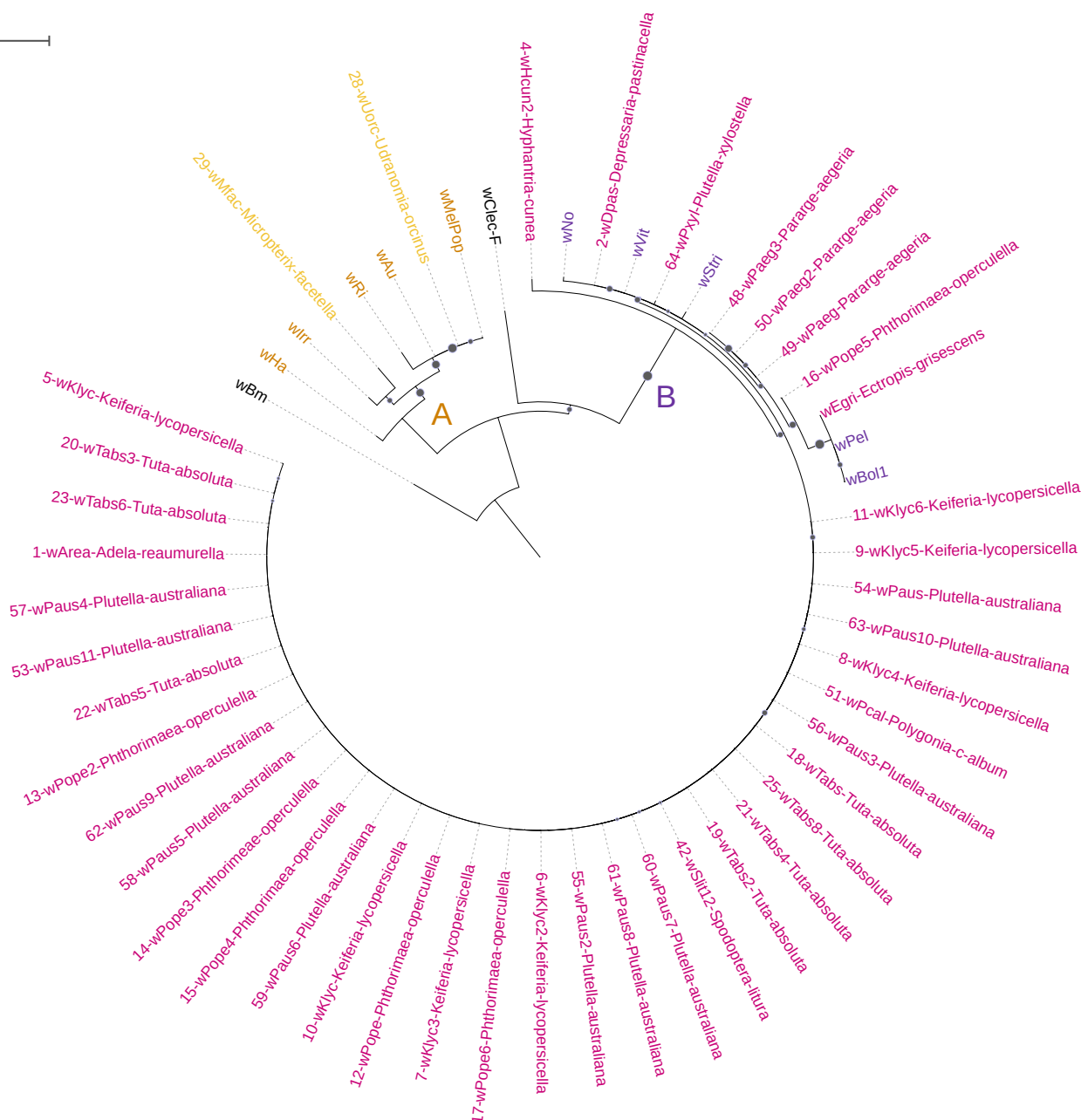

Supplement: Supplementary file 11 — Additional file 11. Figure S5: Phylogenetic relationships between Wolbachia positive SRAs and reference sequences for the MLST gene GatB. [file 12866_2022_2602_MOESM11_ESM.pdf]

Tree scale: 0.1

**bootstrap**

- 0
- 25
- 50
- 75
- 100

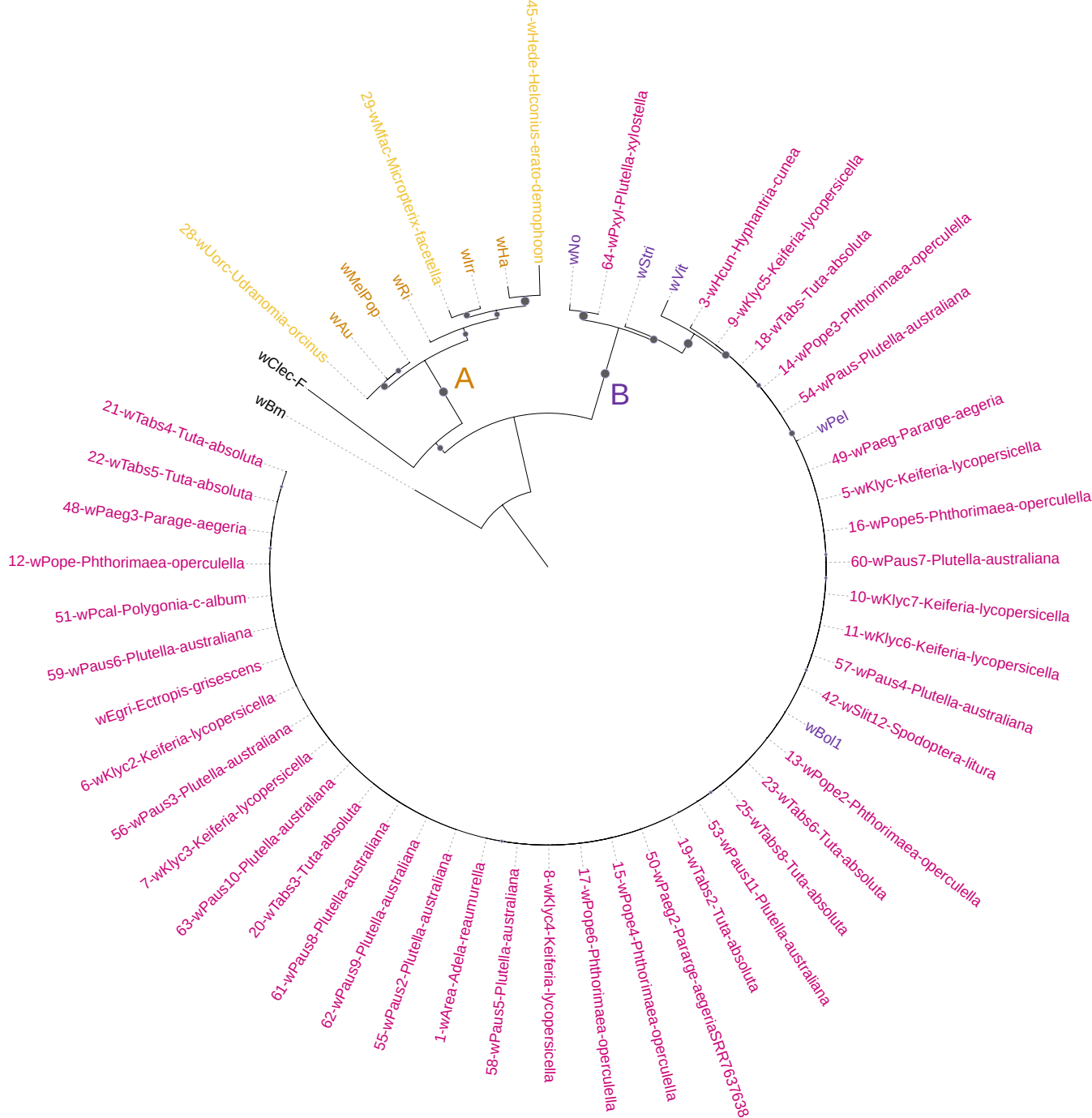

Supplement: Supplementary file 12 — Additional file 12. Figure S6: Phylogenetic relationships between Wolbachia positive SRAs and reference sequences for the MLST gene hcpa. [file 12866_2022_2602_MOESM12_ESM.pdf]

Tree scale: 0.1

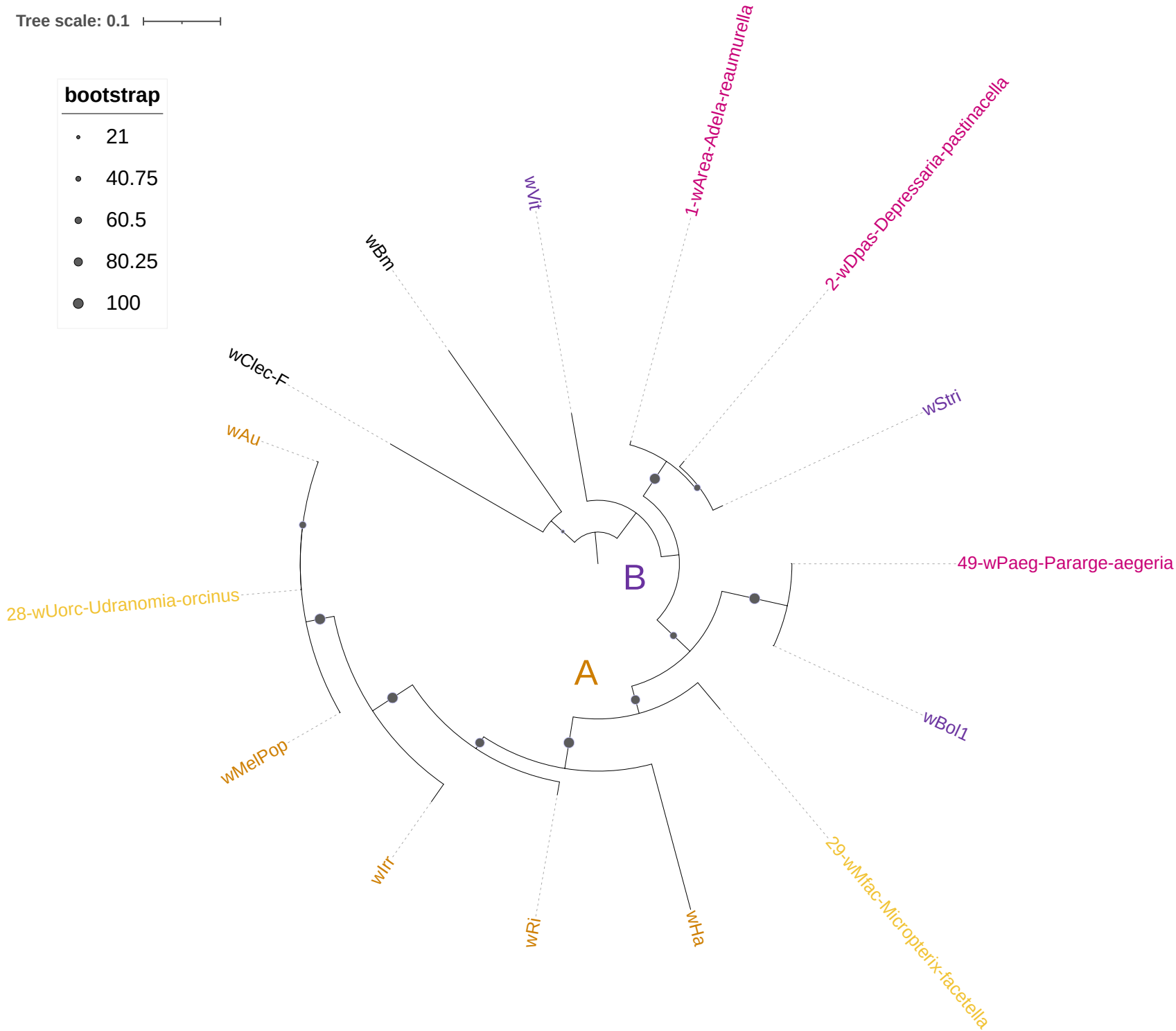

Supplement: Supplementary file 13 — Additional file 13. Figure S7: Phylogenetic relationships between Wolbachia positive SRAs and reference sequences for the wsp gene. [file 12866_2022_2602_MOESM13_ESM.pdf]
